# Multi-scale topology and position feature learning and relationship-aware graph reasoning for prediction of drug-related microbes

**DOI:** 10.1093/bioinformatics/btae025

**Published:** 2024-01-25

**Authors:** Ping Xuan, Jing Gu, Hui Cui, Shuai Wang, Nakaguchi Toshiya, Cheng Liu, Tiangang Zhang

**Affiliations:** School of Computer Science and Technology, Heilongjiang University, Harbin 150080, China; Department of Computer Science, Shantou University, Shantou 515063, China; School of Computer Science and Technology, Heilongjiang University, Harbin 150080, China; Department of Computer Science and Information Technology, La Trobe University, Melbourne, VIC 3083, Australia; School of Information Science and Engineering, Yanshan University, Qinhuangdao 066004, China; Center for Frontier Medical Engineering, Chiba University, Chiba 2638522, Japan; Department of Computer Science, Shantou University, Shantou 515063, China; School of Computer Science and Technology, Heilongjiang University, Harbin 150080, China; School of Mathematical Science, Heilongjiang University, Harbin 150080, China

## Abstract

**Motivation:**

The human microbiome may impact the effectiveness of drugs by modulating their activities and toxicities. Predicting candidate microbes for drugs can facilitate the exploration of the therapeutic effects of drugs. Most recent methods concentrate on constructing of the prediction models based on graph reasoning. They fail to sufficiently exploit the topology and position information, the heterogeneity of multiple types of nodes and connections, and the long-distance correlations among nodes in microbe–drug heterogeneous graph.

**Results:**

We propose a new microbe–drug association prediction model, NGMDA, to encode the position and topological features of microbe (drug) nodes, and fuse the different types of features from neighbors and the whole heterogeneous graph. First, we formulate the position and topology features of microbe (drug) nodes by *t*-step random walks, and the features reveal the topological neighborhoods at multiple scales and the position of each node. Second, as the features of nodes are high-dimensional and sparse, we designed an embedding enhancement strategy based on supervised fully connected autoencoders to form the embeddings with representative features and the more discriminative node distributions. Third, we propose an adaptive neighbor feature fusion module, which fuses features of neighbors by the constructed position- and topology-sensitive heterogeneous graph neural networks. A novel self-attention mechanism is developed to estimate the importance of the position and topology of each neighbor to a target node. Finally, a heterogeneous graph feature fusion module is constructed to learn the long-distance correlations among the nodes in the whole heterogeneous graph by a relationship-aware graph transformer. Relationship-aware graph transformer contains the strategy for encoding the connection relationship types among the nodes, which is helpful for integrating the diverse semantics of these connections. The extensive comparison experimental results demonstrate NGMDA’s superior performance over five state-of-the-art prediction methods. The ablation experiment shows the contributions of the multi-scale topology and position feature learning, the embedding enhancement strategy, the neighbor feature fusion, and the heterogeneous graph feature fusion. Case studies over three drugs further indicate that NGMDA has ability in discovering the potential drug-related microbes.

**Availability and implementation:**

Source codes and Supplementary Material are available at https://github.com/pingxuan-hlju/NGMDA.

## 1 Introduction

The human microbiome is a collection of all microbiota that reside in or on human organs, including bacteria, viruses, protists, fungi, and archaea. Previous human microbiome studies demonstrated that interactions between the human microbes and corresponding hosts regulate human health, such as controlling immune function, providing resistance to pathogens, and even influencing brain physiology and behavior (Duvallet *et al.* 2017, Zhu *et al.* 2020). An imbalance of human microbiota and some diseases are closely related, including chronic inflammation, neurological disorders, and breast cancer (Wang *et al.* 2019, Rackaityte and Lynch 2020).

Microbes can change the toxicity and inhibitory activity of drugs (Nejman *et al.* 2020, Algavi and Borenstein 2023) and impact the effectiveness of disease treatments by biologically altering a drug’s chemical structure (Yin *et al.* 2022). Hacioglu *et al.* (2019) suggested that cooperation between Staphylococcus aureus and Candida albicans leads to drug resistance by strengthening biofilm formation. Also, the gut microbiome produces large quantities of bacterial enzymes that affect therapeutic efficacy (Zimmermann *et al.* 2019). Therefore, discovering new microbe–drug associations is essential in drug functional studies and precision medicine.

Recently, the computational methods were proposed for predicting the drug–target interactions (Li *et al.* 2022), incRNA–miRNA interactions (Wang *et al.* 2022), miRNA–disease associations (Peng *et al.* 2022a), metabolite–disease associations (Gao *et al.* 2023), and incRNA–disease associations (Wang *et al.* 2023a). Computational methods have also shown the ability to determine potential microbe–drug associations and identify reliable drug-related candidates for wet experiments. Microbe–drug association probabilities can be inferred by prediction models using Conditional Random Field (CRF) and Graph Convolutional Network (GCN) (Long *et al.* 2020a). Long *et al.* (2020b) proposed EGTMDA to learn node features for microbes and drugs using meta-paths and hierarchical attention mechanism. SCSMDA enhanced representations of drugs and microbes using graph contrastive learning and elaborate meta-paths (Tian *et al.* 2023). However, shortcomings exist in these methods. GCNMDA used vanilla homogeneous models to learn representations of drugs and microbes without considering abundantly available heterogeneous information. In addition, these methods based on meta-paths focus on neighbors originating from meta-paths while ignoring other non-neighboring nodes across the entire heterogeneous graph.

We proposed NGMDA to predict candidate microbes for drugs by learning the features of drugs and microbes from neighbors and the whole heterogeneous graph. Our contributions are summarized as follows:

- The multi-scale topology information of nodes reflects neighbor regions of different ranges, which is important for microbe–drug association prediction. Therefore, topology features of microbe (drug) nodes are designed based on *t*-step random walks to obtain multi-scale topological neighborhoods of nodes. We also extracted node position features to form the position of each node in the entire heterogeneous graph.
- An embedding enhancement strategy (ES) based on fully connected autoencoders with node class labels is proposed to extract important low-dimensional features of the microbe or drug nodes. This strategy also enhances the differences of feature distributions among different types of nodes by determining the node class.
- In the microbe–drug heterogeneous graph, different neighbor nodes often have special topological neighborhoods and positional features that affect the importance of neighbors with a target node. A new position-sensitive and topology-sensitive self-attention mechanism (PTA) adaptively distinguishes the contributions of different neighbor nodes. Also, neighbor feature fusion (NFF) models heterogeneity of the graph and aggregates the representations of nodes based on heterogeneous graph neural networks (HGNN) with PTA.
- A microbe or drug node may be closely related to distant nodes due to the heterogeneity of the microbe–drug graph. We have designed GFF based on a relationship-aware graph transformer (RAGT) to reveal the diverse connections between the target node and all other nodes in the heterogeneous graph. Comprehensive experiments suggest the superiority of NGMDA by comparing it with advanced methods.

## 2 Materials and methods

**Figure 1.**
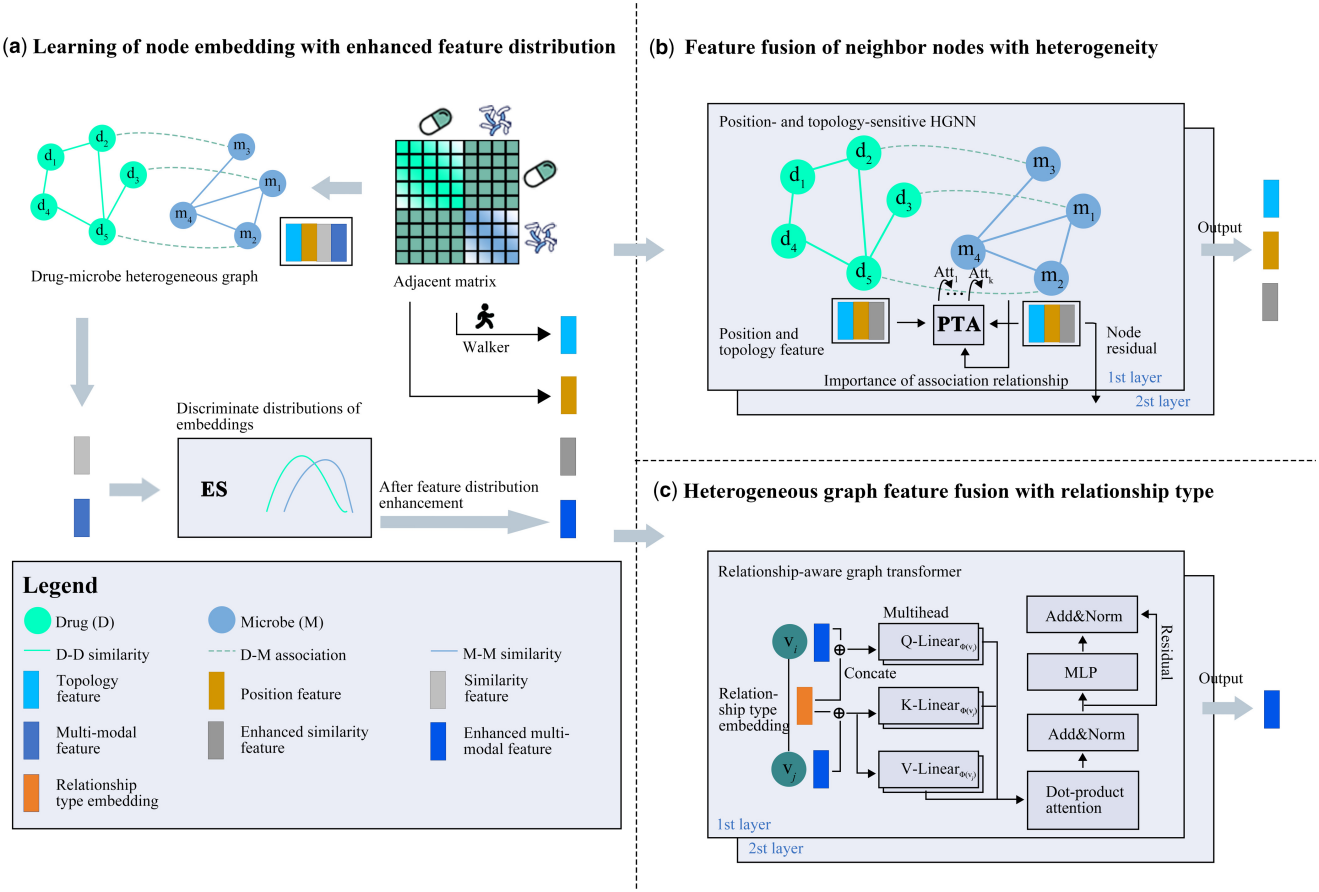
Framework of the proposed NGMDA model. (a) Construct microbe–drug heterogeneous graph and enhance similarity and multi-modal embeddings. (b) Fuse features of neighbor nodes by position- and topology-sensitive HGNN. (c) Learn long-distance connection from the entire heterogeneous graph based on RAGT.

We propose a microbe–drug association prediction model called NGMDA (Fig. 1) that consists of an embedding ES, NFF module, and heterogeneous graph feature fusion (GFF) module. A heterogeneous graph is constructed to describe the diverse connectivity relationships between drugs and microbes (Fig. 1a). The node features of these drugs and microbes are projected into a low-dimensional feature space, and their differences are enhanced to obtain a fine node embedding (Fig. 1a). NFF learns similarity, position, and topology representations between nodes based on position- and topology-sensitive HGNN (Fig. 1b). We use GFF to learn multi-modal representations of various nodes across the heterogeneous graph by a RAGT (Fig. 1c). These four representations are combined into fully connected layers to predict microbe–drug association probabilities.

### 2.1 Dataset

Associations between drugs and microbes, similarities between drugs, and the attribute features of the microbes $X^{\mathrm{micr}}$ are collected from previously published microbe–drug association prediction work (Long *et al.* 2020b). We extracted 2470 microbe–drug association data from the Microbe–Drug Associations Database (MDAD) (Sun *et al.* 2018), which contains 173 microbes and 1373 drugs. Drugbank (Knox *et al.* 2024) provides the interactions among the drugs. On the basis of the biological hypothesis that the drugs with similar treatment functions are more likely interact with the similar microbes, EGTMDA calculated the Gaussian kernel similarities of drugs based on their interactions. The structural similarity of two drugs was measured based on the common subgraphs within their chemical structures (Hattori *et al.* 2010). The final drug similarities were obtained by the weighted sum of the drug Gaussian kernel similarities and the drug structure similarities. The sequences of microbes were extracted from NCBI database, and then principal component analysis was utilized to obtain their important features.

### 2.2 Calculation of microbe similarity

As two microbes with similar gene sequences are typically similar, we calculate the cosine similarity on the attribute characteristics for each microbe. The similarity between microbe $m_{i}$ and $m_{j}$ is $K_{ij}^{\mathrm{micr}}\in [0,1]$,

(1) $$K_{ij}^{\mathrm{micr}}=\frac{1}{2}(\frac{X_{i}^{\mathrm{micr}}{\left(X_{j}^{\mathrm{micr}}\right)}^{T}}{\left\|X_{i}^{\mathrm{micr}}\right\|\left\|X_{j}^{\mathrm{micr}}\right\|}+1),$$

where $X_{i}^{\mathrm{micr}}$ is the i-th row of $X^{\mathrm{micr}}$, which contains the main gene sequence characteristics of $m_{i}$, and ${\left(X\right)}^{T}$ is a transposition of X. The microbe similarities were listed in the Supplementary File SF1.

### 2.3 Microbe–drug heterogeneous graph

We constructed a microbe–drug heterogeneous graph G=(V,E) as shown in Fig. 1a. The node set *V* consists the drug node subset $V^{\mathrm{drug}}$ and microbe node subset $V^{\mathrm{micr}}$ and <i,j>∈E represents an edge from node $v_{j}$ to $v_{i}$. The drug similarity matrix and drug–microbe association matrix are expressed as $K^{\mathrm{drug}}$ and $B^{\mathrm{bipa}}\in R^{N_{d}\times N_{m}}$, respectively, where $N_{d}$ (or $N_{m}$) denotes the number of drugs (or microbes). If there is a known association exists between drugs $d_{i}$ and $m_{j}$, then $B_{i,j}^{\mathrm{bipa}}=1$. Further, $B_{i,j}^{\mathrm{bipa}}=0$ indicates that no connection has yet been found. There are many low similarity data in the similarity matrix, which might be noise in microbe–drug association prediction. When constructing microbe–microbe (or drug–drug) adjacent matrix, connecting edges are added between the microbe (or drug) nodes with a similarity not less than a threshold β. The adjacency matrix of the heterogeneous graph *G* is represented as $B^{\mathrm{hete}}\in R^{\left(N_{d}+N_{m})\times (N_{d}+N_{m}\right)}$, such that

(2) $$B^{\mathrm{hete}}=\left[\begin{array}{cc} {\tilde{K}}^{\mathrm{drug}} & B^{\mathrm{bipa}}\\ {\left(B^{\mathrm{bipa}}\right)}^{T} & {\tilde{K}}^{\mathrm{micr}} \end{array}\right],$$

where ${\tilde{K}}^{\mathrm{drug}}$ (or ${\tilde{K}}^{\mathrm{micr}}$) is the drug (or microbe) similarity matrix after thresholding.

### 2.4 Heterogeneous graph node feature construction and enhancement

#### 2.4.1 Heterogeneous graph node feature construction

The heterogeneous graph node features are constructed by a drug–drug similarity matrix, microbe–microbe similarity matrix, and drug–microbe association matrix. The similarity feature matrix is formed by combining the drug and microbe similarities defined above as

(3) $$H_{\mathrm{simi}}=\left[\begin{array}{c} K^{\mathrm{drug}}\\ K^{\mathrm{micr}} \end{array}\right],$$

where $K_{i}^{\mathrm{drug}}$ (or $K_{j}^{\mathrm{micr}}$) contains the similarities between $d_{i}$ (or $m_{j}$) and other drugs (or microbes). The multi-modal feature matrix $H_{\mathrm{moda}}\in R^{\left(N_{d}+N_{m})\times (N_{d}+N_{m}\right)}$ can be represented as

(4) $$H_{\mathrm{moda}}=\left[\begin{array}{cc} K^{\mathrm{drug}} & B^{\mathrm{bipa}}\\ {\left(B^{\mathrm{bipa}}\right)}^{T} & K^{\mathrm{micr}} \end{array}\right],$$

where the *i*-th row in $H_{\mathrm{moda}}$ records the similarities between $d_{i}$ and all other drugs and the associations between $d_{i}$ and all other microbes. The association with drugs and similarities between microbes are contained in the ($N_{d}+j$)-th row. Because similarity and multi-modal features are common node attributes for microbe–drug association prediction (Peng *et al.* 2017, 2021, Meng *et al.* 2023, Wang *et al.* 2023), we designate these as the original features of the nodes. Existing GNN models fail to fully consider the position and topology information of nodes, so we construct position and topology features of the microbe and drug nodes. The position of $v_{i}$ within the heterogeneous graph is determined by the connection between $v_{i}$ and other nodes. The position feature matrix is defined as $H_{\mathrm{posi}}=B^{\mathrm{hete}}$, where the position feature of $v_{i}$ is $H_{\mathrm{posi},i}$. A random walk of *t*-steps contains a *t*-hop topological neighborhood of nodes within a heterogeneous graph (Dwivedi *et al.* 2022) and is defined as

(5) $$RW^{t}={\left(B^{\mathrm{hete}}{\left(D^{\mathrm{hete}}\right)}^{-1}\right)}^{t},$$

where *t* is the number of walking steps and $D^{\mathrm{hete}}$ is degree matrix of $B^{\mathrm{hete}}$. $RW_{i,j}^{t}$ represents the probability of visiting $v_{i}$ to $v_{j}$ in the *t*-th step random walk and contains the topological neighborhood information of the *t*-th step of $v_{i}$. The topology feature $H_{\mathrm{topo},i}\in R^{t}$ of $v_{i}$ is defined as

(6) $$H_{\mathrm{topo},i}=[RW_{i,i},RW_{i,i}^{2},\ldots ,RW_{i,i}^{t}],$$

which contains the multi-scale topological neighborhood information of $v_{i}$.

#### 2.4.2 Enhancing node embedding

**Figure 2.**
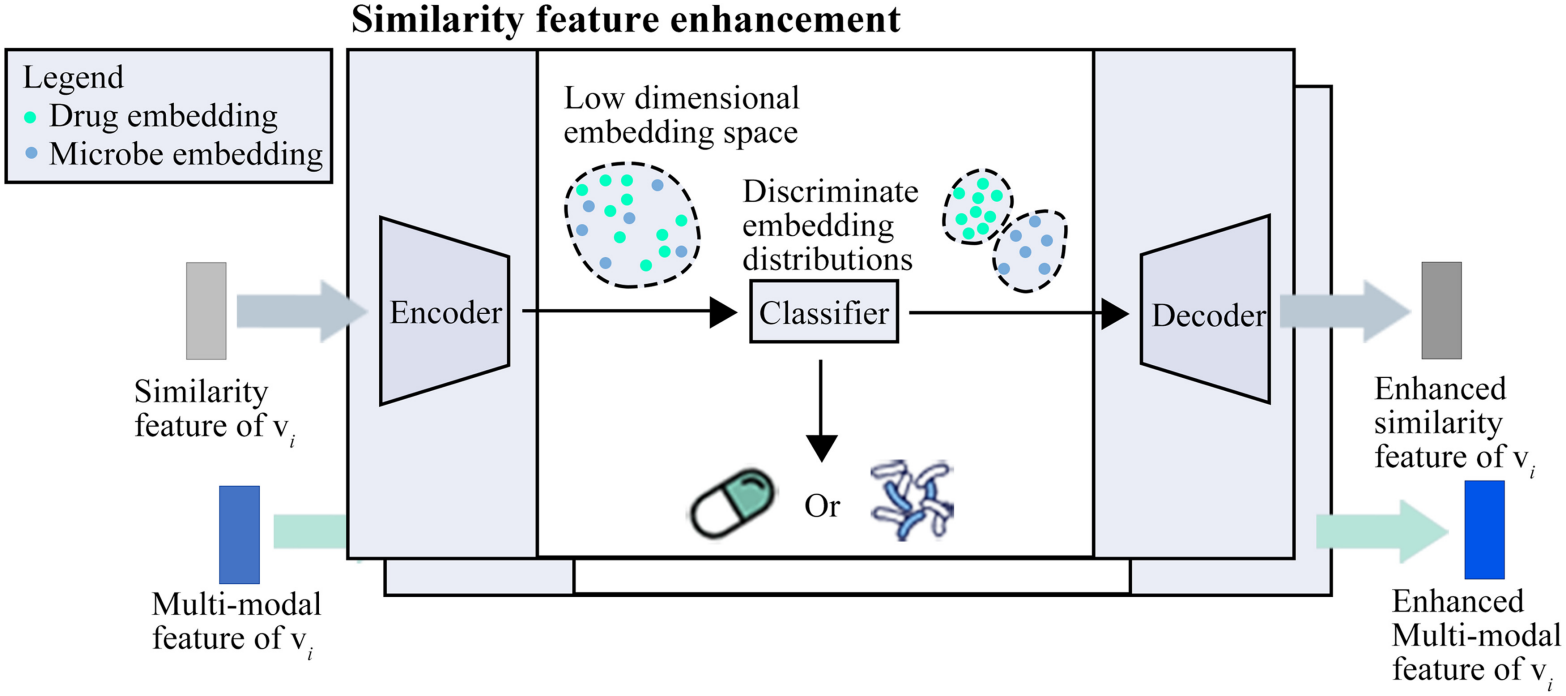
Enhancing node embeddings of microbes and drugs by supervised autoencoders.

The original features specified above are high-dimensional sparse and contain some noise. A projection operation maps drug and microbe node features into the same embedding space, which drops information about the differences in the embedding distributions of different types of nodes. Figure 2 outlines our node embedding ES to learn representative embeddings and enhance the embedding distribution differences of the microbe and drug nodes. As autoencoders could effectively reduce the noise component in these embeddings, we learn important low-dimensional node embeddings based on fully connected autoencoders. The projection and reconstruction process of multi-modal and similarity features are similar, and we use similarity features as an example to describe the process here. The similarity feature of $v_{i}$, $H_{\mathrm{simi},i}$, is projected into $N_{p}$ dimensional space to form

(7) $$H_{\mathrm{simi},i}^{\mathrm{enco},1}=\sigma (\mathrm{Linear}\_\mathrm{enc}o_{\mathrm{simi},\phi (v_{i})}^{\left(1\right)}(H_{\mathrm{simi},i})),$$

where Linear_enco is a linear layer, σ represents the non-linear activation function ReLU, and $\phi (v_{i})$ indicates the type of $v_{i}$. The similarity embedding of $v_{i}$ is learned from the *l*-th fully connected encoding layer as

(8) $$\begin{array}{c} H_{\mathrm{simi},i}^{\mathrm{enco},l}=\sigma (\mathrm{Linear}\_\mathrm{enc}o_{\mathrm{simi},\phi (v_{i})}^{\left(l\right)}(H_{\mathrm{simi},i}^{\mathrm{enco},l-1})),\\ l=1,2,\ldots ,L_{\mathrm{enco}}, \end{array}$$

where $L_{\mathrm{enco}}$ is the total number of encoding layers. $H_{\mathrm{simi},i}^{\mathrm{enco},L_{\mathrm{enco}}}$ is used as the input of the decoder, and the output of the *l*-th fully connected decoding layer is

(9) $$\begin{array}{c} H_{\mathrm{simi},i}^{\mathrm{deco},l}=\sigma (\mathrm{Linear}\_\mathrm{dec}o_{\mathrm{simi},\phi (v_{i})}^{\left(l\right)}(H_{\mathrm{simi},i}^{\mathrm{deco},l-1})),\\ l=1,2,\ldots ,L_{\mathrm{deco}}, \end{array}$$

where $L_{\mathrm{deco}}$ is total number of decoding layers, Linear_deco denotes the linear layer and $H_{\mathrm{simi},i}^{\mathrm{deco},0}=H_{\mathrm{simi},i}^{\mathrm{enco},L_{\mathrm{enco}}}$. After projection, the multi-modal embedding $H_{\mathrm{moda}}^{\mathrm{enco},L_{\mathrm{enco}}}\in R^{\left(N_{d}+N_{m})\times (N_{p}\right)}$ can be learned. The mean square error estimates the reconstruction loss of the node similarity features as

(10) $$\gamma_{\mathrm{reco},\mathrm{simi}}=\frac{1}{\left|T\right|}\sum_{i\in T}{\left\|H_{\mathrm{simi},i}-H_{\mathrm{simi},i}^{L_{\mathrm{deco}}}\right\|}^{2},$$

where *T* is the batch of nodes in the training set. Similarly, the reconstruction loss of the multi-modal feature is $\gamma_{\mathrm{reco},\mathrm{moda}}$.

We classify the projected node embedding to enhance the differences between the drug and microbe embedding distributions. Considering a multi-modal embedding, as an example, is the input of the classify and $\mathrm{labe}l_{i}$ is the corresponding classification labels. The classification loss of the multi-modal embedding in the training samples is estimated by the cross-entropy loss function

(11) $$\gamma_{\mathrm{clas},\mathrm{moda}}=\frac{1}{\left|T\right|}\sum_{i\in T}\sum_{k=1}^{2}-l\mathrm{abe}l_{i,k}\cdot \log(\mathrm{Linear}\_\mathrm{cla}s^{\mathrm{moda}}{\left(H_{\mathrm{moda},i}^{\mathrm{enco},L_{\mathrm{enco}}}\right)}_{k}),$$

where $\mathrm{Linear}\_\mathrm{cla}s^{\mathrm{moda}}\in R^{N_{p}\times 2}$. The classification loss of the similarity embedding is represented as $\gamma_{\mathrm{inty},\mathrm{simi}}$. The total loss of the embedding classification of the drug and microbe nodes is

(12) $$\gamma_{\mathrm{proc}}=\gamma_{\mathrm{reco},\mathrm{simi}}+\gamma_{\mathrm{reco},\mathrm{moda}}+\gamma_{\mathrm{inty},\mathrm{simi}}+\gamma_{\mathrm{inty},\mathrm{moda}}.$$

### 2.5 Neighbor feature fusion

**Figure 3.**
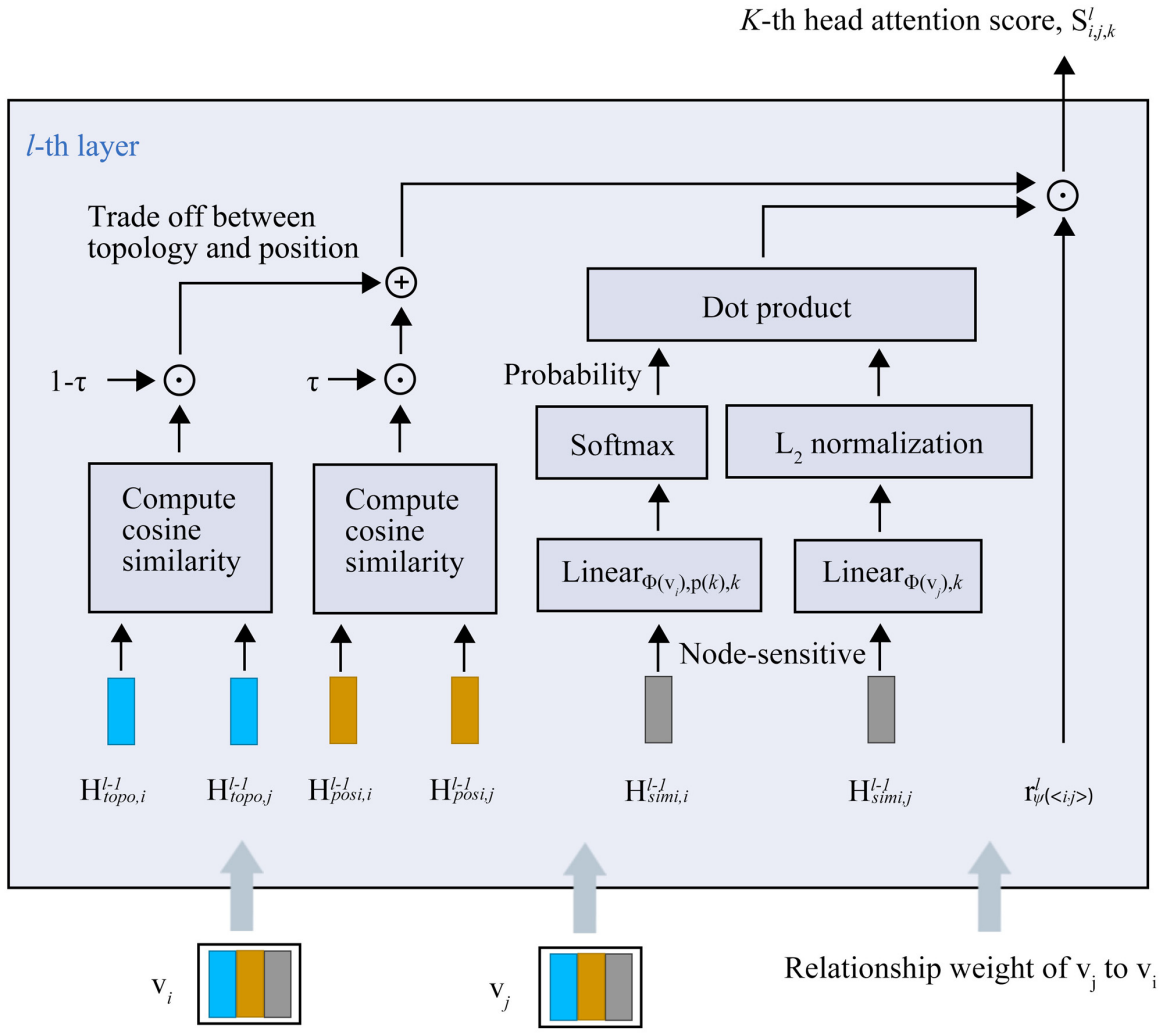
Calculation of position-sensitive and topology-sensitive self-attention of $v_{j}$ to $v_{i}$.

The topological neighborhood and position information of the neighboring nodes impact their importance with a target node. We propose a NFF module based on HGNN with a PTA to learn representative similarity and the position and topology representations of each microbe and drug nodes. The relationship types between the nodes are critical auxiliary features, so we also calculate the importance of the integrated features, as shown in Fig. 3.

The type of relationship between nodes contains the similarity relationship between drugs (or microbes) and the association relationship between drugs (or microbes) and microbes (or drugs). The relationship type of $v_{j}$ to $v_{i}$ is represented as

(13) $$\psi (<i,j>)=\{\begin{array}{ll} 0, & if\;v_{i},v_{j}\in V^{\mathrm{drug}}\\ 1, & if\;v_{i}\in V^{\mathrm{micr}},v_{j}\in V^{\mathrm{drug}}\\ 2, & if\;v_{i}\in V^{\mathrm{drug}},v_{j}\in V^{\mathrm{micr}}\\ 3, & if\;v_{i},v_{j}\in V^{\mathrm{micr}} \end{array}.$$

Then, the importance of the relationship type of $v_{j}$ to $v_{i}$ is $r_{\psi (<i,j>)}^{l}$, which is learned during the training process at the *l*-th layer, and $r_{\psi (<i,j>)}^{0}=1$.

Multi-head attention can reasonably stabilize the learning process of self-attention by allocating the attention value of each head (Veličković *et al.* 2018). After obtaining the similarity representations $H_{\mathrm{simi},j}^{l-1}$ and $H_{\mathrm{simi},i}^{l-1}$ of $v_{i}$ and $v_{j}$, respectively, at l−1-th layer, we compute the importance of the similarity representation of $v_{j}$ to $v_{i}$ in the next layer by

(14) $$\begin{array}{c} s_{i,j,k}^{l}={\left(\mathrm{softmax}(W_{p(k)}^{l}W_{\phi (v_{i}),k}^{l}H_{\mathrm{simi},i}^{l-1})\right)}^{T}n\mathrm{orm}(W_{\phi (v_{j}),k}^{l}H_{\mathrm{simi},j}^{l-1}),\\ l=1,2,\ldots ,L_{\mathrm{nff}}\;\mathrm{and}\;k=1,2,\ldots ,K_{\mathrm{nff}}, \end{array}$$

where $L_{\mathrm{nff}}$ is the total layer number, $K_{\mathrm{nff}}$ is the head number, $H_{\mathrm{simi},i}^{0}=H_{\mathrm{simi},i}^{\mathrm{enco},L_{\mathrm{enco}}}$, $W_{\phi (v_{i}),k}^{l}$ and $W_{p(k)}^{l}\in R^{N_{p}\times N_{p}}$ are weight matrices, and norm represents $L_{2}$ normalization. The $H_{\mathrm{simi},i}^{l-1}$ and $H_{\mathrm{simi},j}^{l-1}$ terms are transformed into latent representations $W_{\phi (v_{i}),k}^{l}H_{\mathrm{simi},i}^{l-1}$ and $W_{\phi (v_{j}),k}^{l}H_{\mathrm{simi},j}^{l-1}$. Then, $W_{p(k)}^{l}W_{\phi (v_{i}),k}^{l}H_{\mathrm{simi},i}^{l-1}$ is the distribution of the similarity representation importance, which is converted to a probability distribution using the softmax function. As the magnitude of the latent representation affects the importance score of $v_{j}$ to $v_{i}$, we standardize $W_{\phi (v_{j}),k}^{l}H_{\mathrm{simi},j}^{l-1}$ with $L_{2}$ normalization. The importance score of $v_{j}$ to $v_{i}$ is calculated by the inner product of the importance distribution of $v_{i}$ and the representation of $v_{j}$. The multiple neighbors of node $v_{i}$ have their various topological neighborhoods and positions, so these neighbors have different importance for $v_{i}$’s feature learning. Therefore, the importance of each neighbor node for $v_{i}$ was calculated before the $v_{i}$’s features were updated. We calculate the importance $c_{i,j}^{l}\in [0,1]$ of the position and topology of $v_{j}$ to $v_{i}$ by

(15) $$c_{i,j}^{l}=[\tau \cdot \mathrm{cosine}(H_{\mathrm{posi},i}^{l-1},H_{\mathrm{posi},j}^{l-1})+(1-\tau )\cdot \mathrm{cosine}(H_{\mathrm{topo},i}^{l-1},H_{\mathrm{topo},j}^{l-1})+1]/2,$$

where $H_{\mathrm{posi},i}^{l-1}$ and $H_{\mathrm{topo},i}^{l-1}$ are the position and topology representations of $v_{i}$, respectively, $H_{\mathrm{posi},i}^{0}=H_{\mathrm{posi},i}$ and $H_{\mathrm{topo},i}^{0}=H_{\mathrm{topo},i}$. The parameter τ∈[0,1] balances the contributions between the position and topology representations. The importance score of $v_{j}$ to $v_{i}$ is then

(16) $$a_{i,j,k}^{l}=\mathrm{softma}x_{j\in N(i)}(r_{\psi (<i,j>)}^{l}\cdot s_{i,j,k}^{l}\cdot c_{i,j}^{l}).$$

Here, $a_{i,j,k}^{l}$ is position and topology sensitive by integrating the importance of the neighbor position and topology.

As residuals can alleviate over-smoothing and vanishing gradients (Lv *et al.* 2021), we add a node residual for every attention head. The similarity representation of $v_{i}$ is then updated as

(17) $$H_{\mathrm{simi},i,k}^{l}=\sigma (\sum_{j\in N(i)}a_{i,j,k}^{l}\cdot W_{\phi (v_{i}),k}^{l}\cdot H_{\mathrm{simi},j}^{l-1}+W_{\mathrm{res}(k)}^{l}\cdot H_{\mathrm{simi},i}^{l-1}),$$

where $W_{\mathrm{res}(k)}^{l}\in R^{N_{p}\times N_{p}}$ is the weight matrix. The similarity representations of the different heads are aggregated at the *l*-th layer to obtain

(18) $$H_{\mathrm{simi},i}^{l}=\frac{1}{K_{\mathrm{nff}}}\sum_{k=1}^{K_{\mathrm{nff}}}H_{\mathrm{simi},i,k}^{l},$$

where $K_{\mathrm{nff}}$ is the head number of the NFF. As the similarity, position, and topology representations have the same update procedure, the position and topology representations are updated as $H_{\mathrm{posi},i}^{l}$ and $H_{\mathrm{topo},i}^{l}$ in the *l*-th layer, respectively.

### 2.6 Heterogeneous GFF

#### 2.6.1 Relationship type encoding

Relationship types of similarity and association can reflect diverse semantic connections between drug and microbe nodes. The relationship type ψ(<i,j>) of $v_{j}$ to $v_{i}$ is represented as a one-hot vector that is linearly transformed to obtain the embedding of the relationship type $e_{\psi (<i,j>)}\in R^{N_{p}}$.

#### 2.6.2 Relationship-aware graph transformer

To capture the connection between the target node and distant nodes, we designed a heterogeneous GFF module presented in Fig. 1c. A RAGT is proposed within the GFF and inspired by these methods (Diao and Loynd 2022, Peng *et al.*2022b, c). To embed the relationship type ψ(<i,j>) of $v_{j}$ to $v_{i}$ into the query, key, and value vectors, we concatenate a multi-modal feature of $v_{i}$ (or $v_{j}$) and the relationship type embedding $e_{\psi (<i,j>)}$. We obtain the query, key, and value vectors of the *h*-th head in the *l*-th layer by linear transformations such that

(19) $$\begin{array}{c} q_{i,j,h}^{l}=[H_{\mathrm{moda},i}^{\mathrm{enco},l-1},e_{\psi (<i,j>)}]W_{\phi (v_{i}),Q,h}^{l},\\ k_{i,j,h}^{l}=[H_{\mathrm{moda},j}^{\mathrm{enco},l-1},e_{\psi (<i,j>)}]W_{\phi (v_{j}),K,h}^{l},\\ v_{i,j,h}^{l}=[H_{\mathrm{moda},j}^{\mathrm{enco},l-1},e_{\psi (<i,j>)}]W_{\phi (v_{j}),V,h}^{l},\\ l=1,2,\ldots ,L_{\mathrm{gff}}\;\mathrm{and}\;h=1,2,\ldots ,K_{\mathrm{gff}}, \end{array}$$

where $W_{\phi (v_{i}),Q,h}^{l},W_{\phi (v_{i}),K,h}^{l},W_{\phi (v_{i}),V,h}^{l}\in R^{2\times N_{p},\frac{N_{p}}{K_{\mathrm{gff}}}}$ are weight matrices, $H_{\mathrm{enc},i}^{G,0}=H_{\mathrm{enc},i}^{G}$, $L_{\mathrm{gff}}$ is the total layer number, and $K_{\mathrm{gff}}$ indicates the head number of GFF. The importance of $v_{j}$ to $v_{i}$ is

(20) $$f_{i,j,h}^{l}=\mathrm{softma}x_{j\in V}(\frac{{\left(q_{i,j,h}^{l}\right)}^{T}k_{i,j,h}^{l}}{\sqrt{N_{p}/K_{\mathrm{gff}}}}).$$

After aggregating the neighbor representations of $v_{i}$ in the *h*-th head, we concatenate the representations from each head to form

(21) $$m_{i}^{l}=\overset{K_{\mathrm{gff}}}{\underset{h=1}{∥}}(\sum_{j=1}^{\left|V\right|}f_{i,j,h}^{l}\cdot v_{i,j,h}^{l}),$$

where ∥ is the concatenation operation. The application of layer normalization (LayerNorm) is crucial for this training process and for expressing the capacity of attention (Brody *et al.* 2023). The multi-modal representation $H_{\mathrm{moda},i}^{l}$ of $v_{i}$ is updated based on LayerNorm, such that

(22) $$\begin{array}{c} n_{i}^{l}=\mathrm{LayerNorm}(W_{1}^{l}m_{i}^{l}+H_{\mathrm{moda},i}^{l-1}),\\ H_{\mathrm{moda},i}^{l}=\mathrm{LayerNorm}(\sigma (W_{2}^{l}n_{i}^{l})W_{3}^{l}+n_{i}^{l}), \end{array}$$

where $W_{1}^{l},W_{2}^{l},W_{3}^{l}\in R^{N_{p}\times N_{p}}$ represent weight matrices.

### 2.7 Representation integration and optimization

The original features and representations learned from shallower layers retain the detailed information of the nodes, along with more abstract information are learned from deeper layers. By concatenating the original features and representations from each layer of NFF and GFF, the final representation of the drug $d_{i}$ is formed as

(23) $${\tilde{H}}_{i}=[H_{\mathrm{simi},i},\overset{L_{\mathrm{nff}}}{\underset{l=0}{∥}}[H_{\mathrm{posi},i}^{l},H_{\mathrm{topo},i}^{l},H_{\mathrm{simi},i}^{l}],H_{\mathrm{moda},i},\overset{L_{\mathrm{gff}}}{\underset{l=0}{∥}}H_{\mathrm{moda},i}^{l}].$$

Likewise, we obtain the final representation ${\tilde{H}}_{j+N_{d}}$ of microbe $m_{j}$. Following the stack of linear layers and the non-linear activation function, ${\tilde{H}}_{i}$ and ${\tilde{H}}_{j+N_{d}}$ are combined to compute the association prediction score $\mathrm{pre}d_{i,j}\in R^{2}$, such that

(24) $$\begin{array}{c} u_{i,j}=\sigma (W_{\mathrm{attr}}[{\tilde{H}}_{i},{\tilde{H}}_{j+N_{d}}]+b_{\mathrm{attr}}),\\ \mathrm{pre}d_{i,j}=\mathrm{softmax}(W_{\mathrm{pred}}u_{i,j}+b_{\mathrm{pred}}), \end{array}$$

where $W_{\mathrm{attr}}$ (or $W_{\mathrm{pred}}$) is the weight matrix and $b_{\mathrm{attr}}$ (or $b_{\mathrm{pred}}$) is a bias vector. Then, $\mathrm{pre}d_{i,j}=[{\left(\mathrm{pre}d_{i,j}\right)}_{0},{\left(\mathrm{pre}d_{i,j}\right)}_{1}]$, where ${\left(\mathrm{pre}d_{i,j}\right)}_{0}$ represents the unrelated probability between $d_{i}$ and $m_{j}$, and the associated probability is ${\left(\mathrm{pre}d_{i,j}\right)}_{1}$. The loss of the association prediction is represented by

(25) $$\gamma_{\mathrm{pred}}=\frac{1}{\left|B\right|}\sum_{(i,j)\in B}\sum_{k=1}^{2}-(\mathrm{label}{\left(i,j\right)}_{k}\cdot \log({\left(\mathrm{pre}d_{i,j}\right)}_{k}),$$

where *B* is the training example set and label(i,j) is the association label of $d_{i}$ and $m_{j}$. The final loss of NGMDA γ is the weighted sum of the ES loss $\gamma_{\mathrm{proc}}$ and the association prediction loss $\gamma_{\mathrm{pred}}$, such that

(26) $$\gamma =\varepsilon \cdot \gamma_{\mathrm{proc}}+(1-\varepsilon )\cdot \gamma_{\mathrm{pred}},$$

where the balance factor ε∈[0,1] is a hyper-parameter.

## 3 Experimental evaluation and discussion

### 3.1 Evaluation metrics

The performance of NGMDA and other comparison methods is evaluated with 5-fold cross-validation. All known associations between drugs and microbes are classified as positive samples, with associations equally divided into five parts. All unobserved microbe–drug associations are taken as negative samples to form a set of negative samples. Four positive examples and an equal number of negative examples are randomly selected from the negative sample to be utilized for training, and the remainder are test examples.

The area under the receiver operating characteristic curve (AUC) (Huang and Ling 2005), the area under the precision–recall curve (AUPR) (Saito and Rehmsmeier 2015), and the recall rate of the top-*k* candidate microbes associated with drugs are selected as our evaluation indicators. If the association score between $d_{i}$ and $m_{j}$ is less than a threshold θ, then it is considered a negative sample. Otherwise, it is identified as a positive sample. The TPRs, FPRs, precisions, and recalls of each drug were calculated at different threshold θ, we calculated the average AUCs and average AUPRs of 1373 drugs for each fold. The 5-fold AUCs (or AUPRs) were averaged as the final AUC (or AUPR). Considering that high-ranking candidates may be chosen by biologists for humidity experiments, more positive samples are expected to appear as top-rank candidates. Hence, we compute a recall rate of the top-*k* candidate microbes of drug $d_{i}$.

### 3.2 Parameter settings

NGMDA runs on a 2080ti server based on the PyTorch framework and is optimized with the Adam algorithm. The proposed model has some hyper-parameters including the steps of random walking, the layer numbers of NFF and that of GFF, and the balance factor of loss ϵ. We firstly establish the variation range for each hyper-parameter, and then select the value which obtains the best performance for the model as the final value of the hyper-parameter. To assess the effect of random walk step size on the prediction performance, the step size was selected from {1, 2, 4, 8, 16, 32}. The model achieves the highest AUC (AUC = 0.944) and AUPR (AUPR = 0.728) when step size is 2 (Supplementary Table ST1). The random walk steps are set to two for the topological embedding formation. For NFF and GFF, we fine-tuned the layer number within a range, {1, 2, 3}, and performed all the combinations of the layer number of NFF and GFF. As shown in the Supplementary Table ST2, the model gets the best performance when their layer numbers are two. The balance factor ε regulates the importance of the loss of embedding ES and that of the association prediction loss, and it was chosen from the range of {0, 0.1, ⋯, 0.5}. Supplementary Table ST3 demonstrates the corresponding results and ε was set to 0.2 finally. The drug (microbe) similarity threshold, β, was selected from {0.5, 0.6, ⋯, 0.9}, and it was set to 0.9 in our experiment (Supplementary Table ST4). Parameter τ is utilized to balance the importance of the topology and position features, and τ varies from 0 to 1 with a step size of 0.2. Supplementary Table ST5 indicates τ value of 0.4 is more favorable for the prediction performance of the model.

### 3.3 Ablation experiments

We perform ablation experiments to evaluate the contributions of position and topology feature learning (PTL), ES, NFF, and GFF as listed in Table 1. For NGMDA without GFF, the AUC and AUPR metrics drop by 1.1% and 6.0%, respectively. The AUC and AUPR of NGMDA without NFF decrease by 1.0% and 5.3%, respectively, compared to the whole model. The AUC and AUPR decrease by 0.6% and 4.6%, respectively, if NGMDA has no ES. The AUC and AUPR of our model achieve 0.9% and 1.5%, respectively, higher than NGMDA without PTL. We built the prediction model without multi-scale topological feature learning and the one without position feature learning, respectively. Their AUCs decreased by 0.8% and 0.4%, and their AUPR decreased by 1% and 0.5%, respectively. After the relationship type integration was eliminated from the prediction model, its AUC and AUPR decreased by 0.6% and 2.7%.

**Table 1.** Results of the ablation studies.

Networks	Average AUC	Average AUPR
NGMDA	0.944	0.728
NGMDA w/o PTL	0.935	0.713
NGMDA w/o ES	0.938	0.682
NGMDA w/o NFF	0.934	0.675
NGMDA w/o GFF	0.933	0.668
NGMDA w/o Topo	0.936	0.718
NGMDA w/o Posi	0.94	0.723
NGMDA w/o Rel	0.938	0.701

The ablation experiments indicate that merging node features of the heterogeneous graph contributes the most to model performance (Table 1). A possible reason is that some non-neighboring nodes exist across the entire heterogeneous graph that are also closely related to the target node. NFF achieves the second most significant contribution, suggesting that the neighboring node information of the target node is also important. The embedding ES boosts the prediction performance, which suggests its value in reducing noise in the node embeddings and enhancing differences in the node distributions. Multi-scale topology and position features indicated the neighbors with multiple ranges and the location information of each node were important for the improved prediction performance. The experimental results (Table 1) also demonstrated the relationship type integration is helpful for improving the prediction performance.

### 3.4 Comparison with other methods

NGMDA is compared with five state-of-the-art microbe–drug association prediction methods, including GCNMDA (Long *et al.* 2020a), EGATMDA (Long *et al.* 2020b), GSAMDA (Tan *et al.* 2022), GACNNMDA (Ma *et al.* 2023), and SCSMDA (Tian *et al.* 2023). NGMDA and five compared methods were trained and tested by using the same data separation during 5-fold cross-validation. The hyper-parameters of these methods are set according to their corresponding literature. We briefly describe these comparison methods in the following.

- GCNMDA (Long *et al.* 2020a): It established a microbe–drug heterogeneous network and integrated multiple kinds of similarities. These similarities were measured based on the chemical structures of drugs, the Gaussian interaction profiles of drugs (microbes), and the microbe sequences. The prediction model was constructed based on GCN and CRF.
- EGATMDA (Long *et al.* 2020b): It constructed a microbe–disease–drug network and then inferred the microbe–drug associations by a hierarchical attention mechanism.
- GSAMDA (Tan *et al.* 2022): The model calculated the drug (microbe) similarities based on the Gaussian interaction profiles and Hamming interaction profiles of drugs (or microbes), and learned the node features by the graph attention networks and sparse auto-encoder.
- GACNNMDA (Ma *et al.* 2023): The multiple microbe–drug heterogeneous networks were constructed based on the Gaussian interaction and Hamming interaction profiles of drugs (microbes). The potential microbe–drug associations were identified by the convolutional neural networks.
- SCSMDA (Tian *et al.* 2023): The model constructed the microbe–drug networks based on the microbe gene sequence information, the Gaussian kernel interaction profiles of drugs (or microbes), and the chemical structures of drugs. It learned the features of the microbe and drug nodes by graph contrastive learning.

We first compute the AUC and AUPR and then calculate the average AUC and AUPR over 1373 drugs. As shown in Table 2, NGMDA achieves the best average AUC of 0.944, which is 0.4% higher than the second-best EGATMDA model, 4.1% better than GCNMDA, 10.1% over GACNNMDA, 4.2% superior to GSAMDA, and 2.8% greater than SCSMDA. NGMDA also produces the best average AUPR of 72.8%, which is 38.8%, 41.3%, 42.1%, 53.2%, and 48.1% better than SCSMDA, GCNMDA, EGATMDA, GACNNMDA, and GASMDA, respectively. We compute the average AUCs (AUPRs) of 1373 drugs per fold for NGMDA and each of the compared methods. To observe whether NGMDA’s prediction performance is significantly higher than each compared method, the statistical test was conducted. NGMDA has 1373 AUCs (AUPRs) for the 1373 drugs, and the compared methods also have 1373 AUCs (AUPRs) for these drugs. The paired Wilcoxon test was executed on NGMDA’s AUCs (AUPRs) and the AUCs (AUPRs) of the compared methods (Table 3). The results indicated NGMDA obtained the significantly higher prediction performance than all the compared methods.

**Table 2.** AUCs and AUPRs of different methods in comparison all the 1373 drugs.

Networks	AUC (%)	AUPR (%)
NGMDA	94.4	72.8
SCSMDA	91.6	34.0
GSAMDA	90.2	24.7
GACNNMDA	84.3	19.6
EGATMDA	94.0	30.7
GCNMDA	90.3	31.5

**Table 3.** The paired Wilcoxon test result on AUCs and AUPRs of 1373 drugs comparing NGMDA with other compared methods.

	GCNMDA	EGATMDA	GACNNMDA	GSAMDA	SCSMDA
*P*-value of AUCs	4.21e-155	1.70e-59	7.26e-159	4.38e-150	1.40e-151
*P*-value of AUPRs	1.47e-186	2.26e-156	6.63e-196	1.30e-186	1.82e-215

The performances of GCNMDA, GACNNMDA, and GSAMDA are not as good as NGMDA, EGATMDA, and SCSMDA. This outcome is likely because these learn node representations using simple models (e.g. GCN and GAT) without considering node or edge types in the microbe–drug heterogeneous graph. EGATMDA and SCSMDA learn the features of drugs and microbes from semantic information based on meta-paths. These models only focus on learning features of the neighbor nodes derived from meta-paths and do not consider the remaining nodes across the entire heterogeneous graph.

**Figure 4.**
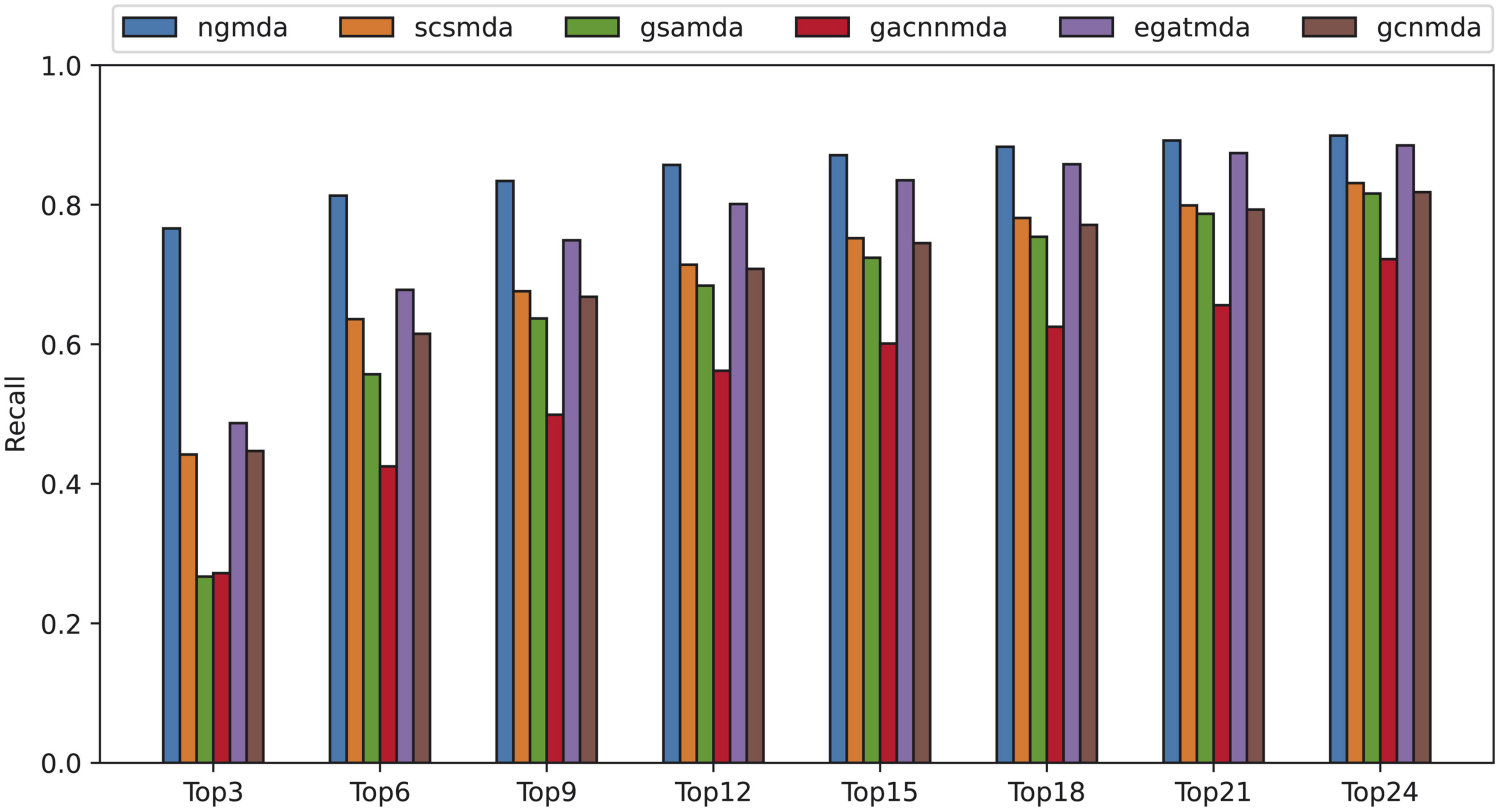
The average recalls of drugs at different top *k* settings.

The average recalls under different top-*k* candidate microbes for all drugs are presented in Fig. 4. NGMDA outperforms all other methods at different top cutoffs due to its enhanced embedding of the nodes and fusing the features of neighbor nodes and the whole heterogeneous graph. When *k *=* *3, our model achieves the highest recall rate of 76.6%, where the second-best 48.7% is attained by EGATMDA. SCSMDA achieves the fourth-best result with a recall rate of 44.7%, which is 0.5% below GCNMDA. When *k* is 6, 9, and 12, NGMDA maintains the best recall values of 81.3%, 83.4%, and 85.7%, respectively. The second performer is EGATMDA with recall rates of 67.8%, 74.9%, and 80.1%, respectively. SCSMDA surpasses GCNMDA with recall rates of 63.6%, 67.6%, and 71.4%, respectively, while the recall rates of GCNMDA are lower at 61.5%, 66.8%, and 70.8%, respectively. GSAMDA does not perform well with recall rates of 55.7%, 63.7%, and 68.4%, respectively, while still being consistently higher than GACNNMDA, which obtained the lowest recall rates of 42.5%, 49.9%, and 56.2%, respectively.

### 3.5 Case studies on three drugs

To confirm NGMDA’s discovery potential of drug-related microbial candidates, case studies with Ciprofloxacin, Moxifloxacin, and Vancomycin are performed. Ciprofloxacin treats skin infections, typhoid fever, pneumonia, endocarditis, and other bacterial infections. Moxifloxacin treats pneumonia, tuberculosis, sinusitis, and chronic bronchitis. Vancomycin is an antibiotic that treats bloodstream infections, endocarditis, and orthopedic infections. All the known microbe–drug associations and the randomly selected equal number of unobserved microbe–drug associations were utilized to train the model for case studies. Candidate microbes are obtained for each of these drugs, and we collected the top 20 candidates, as listed in Tables 4–6.

**Table 4.** The top-20 candidate microbes of Ciprofloxacin.

Rank	Microbe name	Evidence	Rank	Microbe name	Evidence
1	*Candida albicans*	PMID: 31471074	11	*Bacillus subtilis*	MDAD
2	*Pseudomonas aeruginosa*	aBiofilm, MDAD	12	*Actinomyces oris*	Unconfirmed
3	*Staphylococcus aureus*	aBiofilm, MDAD	13	*Human immunodeficiency virus 1*	PMID: 9566552
4	*Escherichia coli*	aBiofilm, MDAD	14	*Streptococcus sanguis*	PMID: 11347679
5	*Streptococcus mutans*	PMID: 30468214	15	*Stenotrophomonas maltophilia*	aBiofilm, MDAD
6	*Staphylococcus epidermis*	PMID: 10632381	16	*Haemophilus influenzae*	MDAD
7	*Staphylococcus epidermidis*	PMID: 28481197	17	*Listeria monocytogenes*	PMID: 28355096
8	*Salmonella enterica*	PMID: 26933017	18	*Burkholderia cenocepacia*	PMID: 27799222
9	*Vibrio harveyi*	PMID: 27247095	19	*Streptococcus pneumoniae*	PMID: 26100702
10	*Enterococcus faecalis*	PMID: 27790716	20	*Serratia marcescens*	PMID: 23751969

**Table 5.** The top-20 candidate microbes of Moxifloxacin.

Rank	Microbe name	Evidence	Rank	Microbe name	Evidence
1	*Pseudomonas aeruginosa*	PMID: 31691651	11	*Staphylococcus epidermidis*	PMID: 11249827
2	*Staphylococcus aureus*	PMID: 31689174	12	*Candida albicans*	aBiofilm, MDAD
3	*Escherichia coli*	PMID: 31542319	13	*Streptococcus pneumoniae*	PMID: 22407042
4	*Vibrio harveyi*	Unconfirmed	14	*Serratia marcescens*	Unconfirmed
5	*Bacillus subtilis*	PMID: 30036828	15	*Acinetobacter baumannii*	PMID: 12951327
6	*Listeria monocytogenes*	PMID: 28739228	16	*Actinomyces oris*	PMID: 26538502
7	*Salmonella enterica*	PMID: 22151215	17	*Clostridium perfringens*	PMID: 29486533
8	*Stenotrophomonas maltophilia*	aBiofilm, MDAD	18	*Klebsiella pneumoniae*	PMID: 27257956
9	*Burkholderia cenocepacia*	PMID: 28355096	19	*Burkholderia pseudomallei*	PMID: 15731198
10	*Burkholderia multivorans*	Unconfirmed	20	*Haemophilus influenzae*	MDAD

**Table 6.** The top-20 candidate microbes of Vancomycin.

Rank	Microbe name	Evidence	Rank	Microbe name	Evidence
1	*Staphylococcus aureus*	MDAD; aBiofilm	11	*Streptococcus mutans*	PMID: 464571
2	*Pseudomonas aeruginosa*	PMID: 26980934	12	*Stenotrophomonas maltophilia*	Unconfirmed
3	*Escherichia coli*	PMID: 33468474	13	*Streptococcus pneumoniae*	PMID: 10376600
4	*Staphylococcus epidermidis*	PMID: 20685088	14	*Acinetobacter baumannii*	PMID: 23422916
5	*Bacillus subtilis*	PMID: 14165485	15	*Actinomyces oris*	PMID: 26538502
6	*Enterococcus faecalis*	PMID: 15528891	16	*Salmonella enterica*	Unconfirmed
7	*Vibrio harveyi*	PMID: 25066453	17	*Klebsiella pneumoniae*	Unconfirmed
8	*Listeria monocytogenes*	PMID: 10588323	18	*Clostridium perfringens*	PMID: 16870765
9	*Burkholderia cenocepacia*	Unconfirmed	19	*Serratia marcescens*	Literature (Ali 2018)
10	*Burkholderia multivorans*	Unconfirmed	20	*Streptococcus sanguis*	PMID: 7287904

The MDAD (Sun *et al.* 2018) provides microbe–drug associations that were verified by experimental or clinical studies. The aBiofilm database (Rajput *et al.* 2018) organizes data on anti-biofilm agents disrupting biofilms, covering 1720 drugs and 140 microbes. We use MDAD, aBiofilm database, and literature to verify the microbe–drug association prediction results of NGMDA. Among the top 20 candidate microbes related to Ciprofloxacin, six are recorded by MDAD, and four are contained in the aBiofilm database, which suggests that these microbes are indeed associated with the drug Ciprofloxacin, and these 13 candidates are further confirmed by the literature. For example, several microbes, including Candida albicans, Human immunodeficiency virus 1, Streptococcus mutans, and Streptococcus pneumoniae, are inhibited (or killed) by Ciprofloxacin (Gollapudi *et al.* 1998, Dridi *et al.* 2015, Hacioglu *et al.* 2019, Zhang *et al.* 2019). The two microbes, Staphylococcus epidermidis and Salmonella enterica, were validated to be highly susceptible to Ciprofloxacin (Eibach *et al.* 2016, Szczuka *et al.* 2017). In addition, Vibrio harveyi, Enterococcus faecalis, and Listeria monocytogenes are identified as Ciprofloxacin-resistant microbes (Stalin and Srinivasan 2016, Escolar *et al.* 2017, Kim and Woo 2017). For the microbe candidates related to Moxifloxacin in Table 5, three candidates are included in MDAD, two in the aBiofilm database and 14 candidates are supported by the literature. Considering the candidate microbes of Vancomycin in Table 6, Staphylococcus is confirmed by the MDAD and aBiofilm databases, and 14 candidates are supported by literature. Among all 60 microbe candidates, nine are unconfirmed, which indicates that no relevant evidence is found to support their association. The above analysis demonstrates that NGMDA can discover potential candidate microbes for target drugs under study.

### 3.6 Prediction of novel microbe–drug associations

NGMDA is implemented to predict the potential candidate microbes for all drugs. The top-ranked 20 microbe candidates are listed in the Supplementary File SF2, which can be leveraged by biologists to screen reliable candidate microbes.

## 4 Conclusion

We proposed a novel microbe–drug association prediction model to encode node neighborhood topologies across multiple scales and perform graph inference by propagating different types of connections and information about the nodes. The multi-scale topology feature is formed by estimating the probability that a random walker accesses itself in different steps. The established node embedding strategy enhances the representations of microbe and drug nodes that form the specific distribution of the corresponding node. The NFF combines the features of different types of neighbors and target nodes by adaptively evaluating the weights of the position features, topology features, and original features of the neighbor nodes. The long-distance connections and encoding of the relationship types between the nodes through GFF enable the knowledge propagation of the entire graph and the capture of diverse relationships. Cross-validation experimental results on public datasets suggest the superiority and effectiveness of NGMDA. The average recall rate of drugs and case analyses of experimental results further demonstrate that NGMDA provides reliable microbe candidates for related drugs under investigation.

## Supplementary Material

btae025_Supplementary_Data

## Data Availability

The dataset is available at https://github.com/pingxuan-hlju/NGMDA.
