# Supplementary material for: Multi-scale topology and position feature learning and relationship-aware graph reasoning for prediction of drug-related microbes
Source: Bioinformatics. 2024 Jan 25;40(2):btae025. doi: 10.1093/bioinformatics/btae025 (PMC10868329; doi:10.1093/bioinformatics/btae025)
Supplement: btae025_Supplementary_Data [file btae025_supplementary_data.zip › Supplement materials.docx]

**Supplementary Materials for “Multi-scale topology and position feature learning and relationship-aware graph reasoning for prediction of drug-related microbes”**

**1 Parameter analysis**

We conduct experiments to measure parameter influence on the proposed model. The results in Table ST1, Table ST2, Table ST3, Table ST4, and Table ST5 indicate that our model achieves better performance when step_size= 2, layer number= 2,ε= 0.2,β= 0.9, and τ= 0.4.

**Supplementary Table ST1.** Prediction performance for the step size of random walk.

| Step size of random walk | AUC | AUPR |
| --- | --- | --- |
| 1 | 0.942 | 0.722 |
| 2 | 0.944 | 0.728 |
| 4 | 0.94 | 0.718 |
| 8 | 0.941 | 0.717 |
| 16 | 0.938 | 0.71 |
| 32 | 0.94 | 0.72 |

**Supplementary Table ST2.** Prediction performance for the layer number of NFF and GFF.

| NFF | GFF | AUC | AUPR |
| --- | --- | --- | --- |
| 1 | 1 | 0.945 | 0.715 |
| 1 | 2 | 0.943 | 0.714 |
| 1 | 3 | 0.946 | 0.72 |
| 2 | 1 | 0.943 | 0.708 |
| 2 | 2 | 0.944 | 0.728 |
| 2 | 3 | 0.948 | 0.722 |
| 3 | 1 | 0.942 | 0.705 |
| 3 | 2 | 0.943 | 0.713 |
| 3 | 3 | 0.945 | 0.724 |

**Supplementary Table ST3.** Prediction performance for the balance factor of loss.

| ε | AUC | AUPR |
| --- | --- | --- |
| 0 | 0.938 | 0.682 |
| 0.1 | 0.946 | 0.712 |
| 0.2 | 0.944 | 0.728 |
| 0.3 | 0.943 | 0.712 |
| 0.4 | 0.94 | 0.703 |
| 0.5 | 0.941 | 0.7 |

**Supplementary Table ST4.** Prediction performance for the threshold of similarity.

| β | AUC | AUPR |
| --- | --- | --- |
| 0.5 | 0.918 | 0.674 |
| 0.6 | 0.923 | 0.703 |
| 0.7 | 0.939 | 0.691 |
| 0.8 | 0.941 | 0.712 |
| 0.9 | 0.944 | 0.728 |

**Supplementary Table ST5.** Prediction performance for the balance factor of position and topology representation.

| τ | AUC | AUPR |
| --- | --- | --- |
| 0 | 0.941 | 0.723 |
| 0.2 | 0.943 | 0.721 |
| 0.4 | 0.944 | 0.728 |
| 0.6 | 0.942 | 0.717 |
| 0.8 | 0.939 | 0.718 |
| 1 | 0.937 | 0.714 |

**2 Case studies**

For further validating the effectiveness of our proposed model, we performed Case study on three drugs (i.e., Ciprofloxacin, Moxifloxacin, and Vancomycin). Table ST6 presents in detail the candidate microbes for Ciprofloxacin. Note that antibacterial activity in description refers to the ability of drugs to inhibit or kill microbes.

**Table ST6**. Candidate microbes of the drug Ciprofloxacin.

| Microbe name | Evidence | Description |
| --- | --- | --- |
| Candida albicans | PMID: 31471074 | Antibacterial activity |
| Pseudomonas aeruginosa | aBiofilm, MDAD | Inhibiting biofilm formation |
| Staphylococcus aureus | aBiofilm, MDAD | Inhibiting biofilm formation |
| Escherichia coli | aBiofilm, MDAD | Inhibiting biofilm formation |
| Streptococcus mutans | PMID: 30468214 | Killing effectiveness |
| Staphylococcus epidermis | PMID: 10632381 | Antibacterial activity |
| Staphylococcus epidermidis | PMID: 28481197 | Antibacterial activity |
| Salmonella enterica | PMID: 26933017 | Ciprofloxacin susceptibility |
| Vibrio harveyi | PMID: 27247095 | Resistance to ciprofloxacin |
| Enterococcus faecalis | PMID: 27790716 | Resistance to ciprofloxacin |
| Human immunodeficiency virus 1 | PMID: 9566552 | Inhibiting microbial activity |
| Streptococcus sanguis | PMID: 11347679 | Inhibiting microbial activity |
| Stenotrophomonas maltophilia | aBiofilm, MDAD | Inhibiting biofilm formation |
| Listeria monocytogenes | PMID: 28355096 | Resistance to ciprofloxacin |
| Burkholderia cenocepacia | PMID: 27799222 | Resistance to ciprofloxacin |
| Streptococcus pneumoniae | PMID: 26100702 | Resistance to ciprofloxacin |
| Serratia marcescens | PMID: 23751969 | Resistance to ciprofloxacin |
